# Supplementary material for: Vitamin D Modified DSS-Induced Colitis in Mice via STING Signaling Pathway
Source: Biology (Basel). 2025 Jun 18;14(6):715. doi: 10.3390/biology14060715 (PMC12190092; doi:10.3390/biology14060715)
Supplement: Supplementary file 1 [file biology-14-00715-s001.zip › Supplementary Materials S1: 16s-report of gut microbiota/Groups/diff_analysis/heatmap/Familyclusterdiffheatmap.pdf]

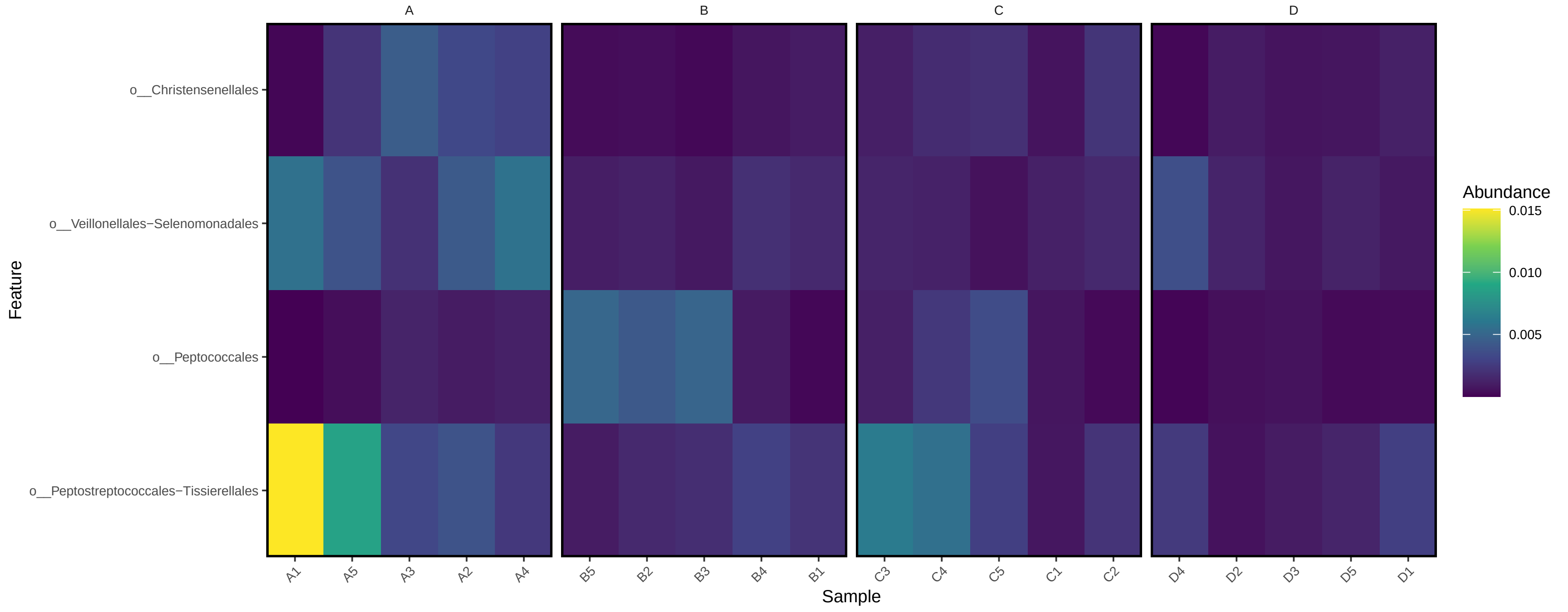

A
B
C
D
o__Christensenellales
o__Veillonellales−Selenomonadales
o__Peptococcales
o__Peptostreptococcales−Tissierellales
Feature
Abundance
0.015
0.010
0.005
A1
A5
A3
A2
A4
B5
B2
B3
B4
B1
C3
C4
C5
C1
C2
D4
D2
D3
D5
D1
Sample
